# Surgical treatment of severe benign tracheal stenosis

**DOI:** 10.1186/s13019-023-02369-0

**Published:** 2023-10-14

**Authors:** Yong-Geng Feng, Shao-Lin Tao, Long-Yong Mei, Fu-Qiang Dai, Qun-You Tan, Ru-Wen Wang, Jing-Hai Zhou, Bo Deng

**Affiliations:** Thoracic Surgery Department, Institute of Surgery Research, Daping Hospital, Army Medical University, Changjiang Branch St, 10#, Yuzhong District, Chongqing city, 400042 China

**Keywords:** Trachea, Benign stenosis, Surgical treatment

## Abstract

**Objective:**

To present clinical experiences regarding surgical treatment of patients with severe cicatricial tracheal stenosis.

**Patients and methods:**

From January 2008 to March 2020, 14 patients underwent tracheal resection and reconstruction under general anesthesia. Nine cases had cervical tracheal stenosis and five cases had thoracic tracheal stenosis. The mean diameter and length of strictured trachea was 0 − 8 mm with a mean of 4.5 ± 2.4 mm and 1 − 3 cm with a mean of 1.67 ± 0.63 cm, respectively. General anesthesia and mechanical ventilation were performed in ten cases and four patients underwent femoral arteriovenous bypass surgery due to severe stenosis. End-to-end anastomosis of trachea was performed in 13 cases and the anastomosis between trachea and cricothyroid membrane was performed in one case. Absorbable and unabsorbable sutures were used for the anterior and posterior anastomoses, respectively. Postoperative neck anteflexion was maintained by a suture between the chin and superior chest wall. The relevant data of the 14 patients were retrospectively reviewed, and the operation time, blood loss, postoperative hospital stay, postoperative complications and follow-up were retrieved.

**Results:**

There was no intraoperative death. The length of resected trachea ranged from 1.5 to 4.5 cm with a mean of 1.67 ± 0.63 cm. Operation time ranged from 50 − 450 min with a mean of 142.8 ± 96.6 min and intraoperative hemorrhage ranged from 10 − 300 ml with a mean of 87.8 ± 83.6 ml. Follow-up period ranged from 5 to 43 months with a mean of 17.9 ± 10.6 months. None of the patients had recurrent laryngeal nerve paralysis during postoperative follow-up. Ten cases were discharged uneventfully. Anastomosis stenosis occurred in three cases who received interventional therapies. Bronchopleurocutaneous fistula occurred in one patient after 6 days postoperatively and further treatment was declined.

**Conclusion:**

The strategies of anesthesia, mechanical ventilation, identification of stenosis lesion, the “hybrid” sutures and postoperative anteflexion are critical to be optimized for successful postoperative recovery.

## Introduction

Benign tracheal stenosis (TS) can be caused by tracheal intubation, incision or trauma. It is estimated to occur in 1.8–12% of long-term invasively ventilated patients [1, 2], but severe TS with respiratory symptoms, such as wheezing and dyspnea, is relatively rare [3–5]. Mild and asymptomatic stenosis of the trachea does not require treatment. Once severe TS occurs, urgent treatment is required to control the life-threatening dyspnea and asphyxia caused by secretion retention and inflammatory edema. Although there are a variety of approaches to treat TS, including bronchoscope balloon dilatation, cryotherapy, laser cauterization of scarring and placement of a stent, surgical resection of cicatricial stenosis and reconstruction of trachea is still the most effective method for treatment [6–8]. The key points of surgical treatment and postoperative recovery are anesthesia, mechanical ventilation, identification of stenosis lesions and relevant strategies of tracheal reconstruction. The complications during surgical treatment include anesthesia and mechanical ventilation (MV), anastomosis without tension and postoperative management of stenosis following anastomosis (AS). From January 2008 to March 2020, there were 14 patients with severe TS who underwent tracheal resection and reconstruction in this hospital and the case management and outcomes are presented.

## Patients and methods

### Patients

The clinical and demographical data of patients who underwent surgical treatment for tracheal stenosis from January 2008 to March 2020 in Daping Hospital, were retrospectively reviewed excluding tracheal stenosis resulting from primary tracheal tumors or radiotherapy due to head and neck neoplasms. Finally, 14 patients met the criteria including 12 males and two females with a mean age of 43.2 ± 13.9 years. Ten cases suffered from injuries including three cases caused by falling, three cases in car accidents, two cases due to tracheal penetrating wounds, one case injured by a heavy falling object and one case with idiopathic chest and abdomen trauma. They were all intubated in emergency and six patients received conventional tracheotomy during subsequent treatment. Among the other four cases, one case underwent tracheal intubation and three cases received conventional tracheotomy due to acute respiratory distress syndrome (ARDS) caused by spontaneous pneumothorax, severe pneumonia, organophosphorus poisoning and hematencephalon.

All the cases underwent initial treatment in their local hospital. However, dyspnea was found in them after tracheal extubation after a mean time of 90.4 ± 102.1 days. One patient received tracheal dilatation twice and one patient received laser cauterization seven times in the local hospital, without relieving the dyspnea.

**Fig. 1:**
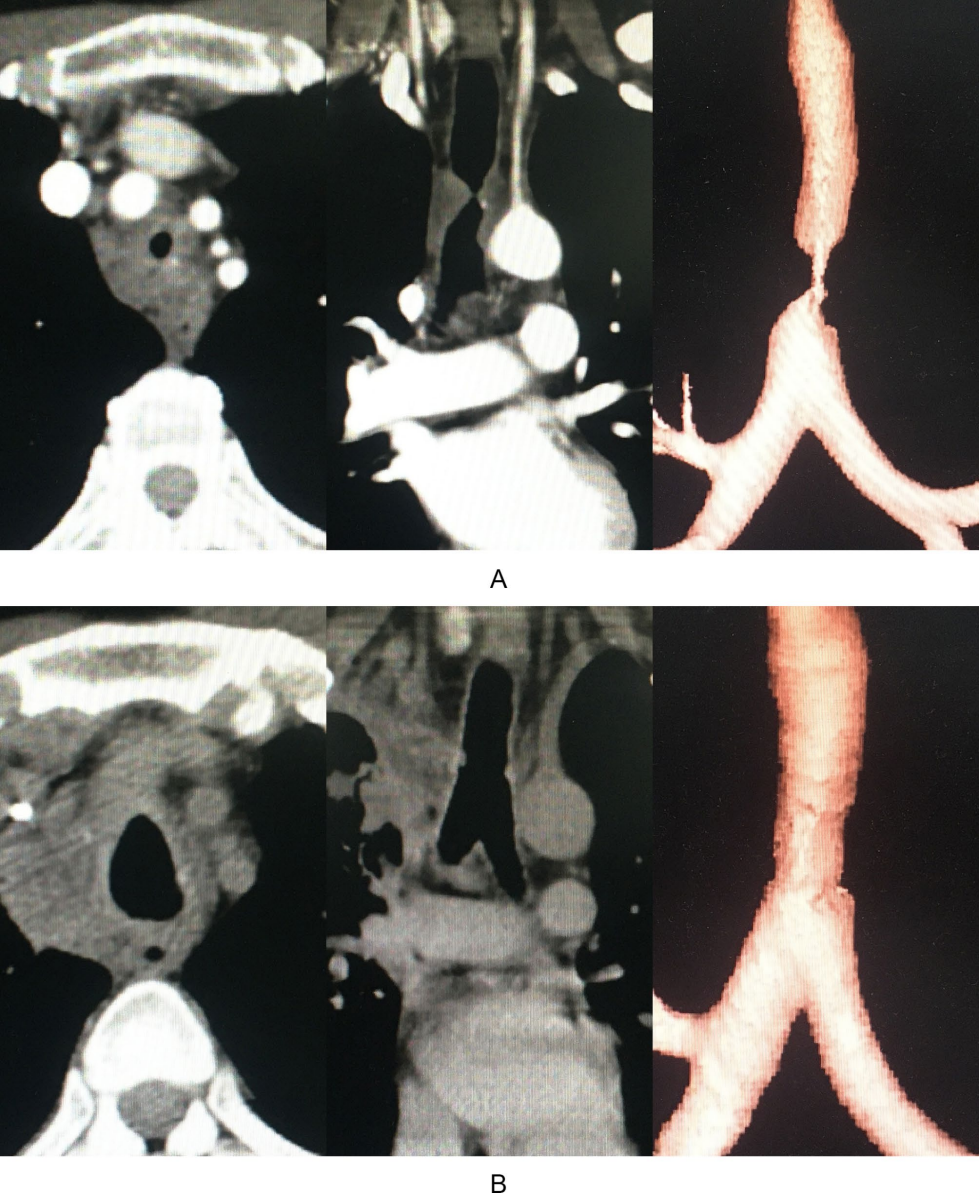
(**A**) CT scan showed the severe TS in case #13 prior to operation; (**B**) CT scan showed the reconstructed trachea

The patients were referred to this institution for further treatment. Nine cases had cervical TS and five had thoracic TS, confirmed by fiberoptic bronchoscopy or CT examination as shown in Fig. 1A. The mean diameter of tracheal stenosis was 0 − 8 mm with a mean of 4.5 ± 2.4 mm and length was 1 − 3 cm with a mean of mean:1.67 ± 0.63 cm. The length of the upper margin of stricture from glottis was 1 − 7 cm with a mean of 3.1 ± 1.9 cm and lower margin to carina was 1.5 to 8.9 cm, with a mean of 5.5 ± 2.2 cm. The Clinical and demographical features of the fourteen cases were shown in Table 1.

**Table 1:** Clinical and demographical features of 14 cases

Case	Gender	Age (years)	Primary disease	Tracheal intubation or tracheotomy	Dyspnea after tracheal extubation (Days)	Intervention treatment before operation	Location of stenosis	Diameter of stenosis(mm)	Length of stenosis(cm)	The length of the upper margin of stenosis from glottis(cm)	The length of the lower margin to carina(cm)
#1	Male	51	Severe pneumonia	Tracheal intubation	66	Oxygen inhalation	Cervical trachea	3	1	3	N.A.
#2	Male	56	Car accidents	Tracheal intubation	49	Oxygen inhalation	Cervical trachea	4	1.4	2	7
#3	Male	25	Falling injury	Tracheotomy	16	Tracheal intubation	Cervical - thoracic trachea	8	2.1	1.5	7.5
#4	Male	22	Spontaneous pneumothorax	Tracheotomy	233	Tracheotomy	Cervical trachea	5	3	5	4.4
#5	Female	46	Car accidents	Tracheal intubation	52	Tracheal intubation	Cervical trachea	5	1.5	1	2.5
#6	Male	30	Hematencephalon	Tracheotomy	24	Tracheal intubation	Thoracic trachea	8	2.5	6	2.7
#7	Male	61	Heavy object injury	Tracheotomy	59	Balloon dilation 2 times	Cervical trachea	4	1.9	3.8	6.6
#8	Male	64	Car accidents	Tracheal intubation	270	Oxygen inhalation	Cervical trachea	6	1	3	6.7
#9	Female	43	Falling injury	Tracheotomy	360	Oxygen inhalation	Cervical - thoracic trachea	4	1.3	3	5.4
#10	Male	60	Falling injury	Tracheotomy	46	Oxygen inhalation	Cervical trachea	4	1.5	1	8.9
#11	Male	45	Tracheal penetrating wounds	Tracheotomy	180	Tracheotomy	Cervical trachea	0	2.5	1	6.5
#12	Male	31	Tracheal penetrating wounds	Tracheotomy	15	Tracheotomy	Cervical trachea	0	1	2.5	7
#13	Male	32	Organophosphorus poisoning	Tracheotomy	150	Laser cauterization 7 times	Thoracic trachea	6	3	7	1.5
#14	Male	39	Idiopathic chest and abdomen trauma	Tracheal intubation	15	Oxygen inhalation	Cervical trachea	6	2.5	4	4.7

This retrospective study was approved by the Ethics Committee of Daping Hospital of Army Medical University NO.185 (2019) which waived the requirement for informed consent and was conducted in accordance with the Declaration of Helsinki and Ethical Guidelines for Medical and Health Research Involving Human Subjects.

### Methods

The status of patients was assessed before anesthesia, and femoral artery-venous bypass surgery was performed in case of intolerance of dilation supported by mask oxygen inhalation. Prior to general anesthesia, mechanical ventilation (MV) was performed through primary tracheostomy or via tracheal catheter which was inserted into proximal end of stenosis guided by fiberoptic bronchoscopy.

Following general anesthesia, a shoulder roll was placed to allow hyperextension of neck in the supine position. The tissues, muscle layers and thyroid isthmus anterior to trachea were separated. To avoid the injury to the recurrent laryngeal nerve, its isolation and TS resection were performed carefully.

**Fig. 2:**
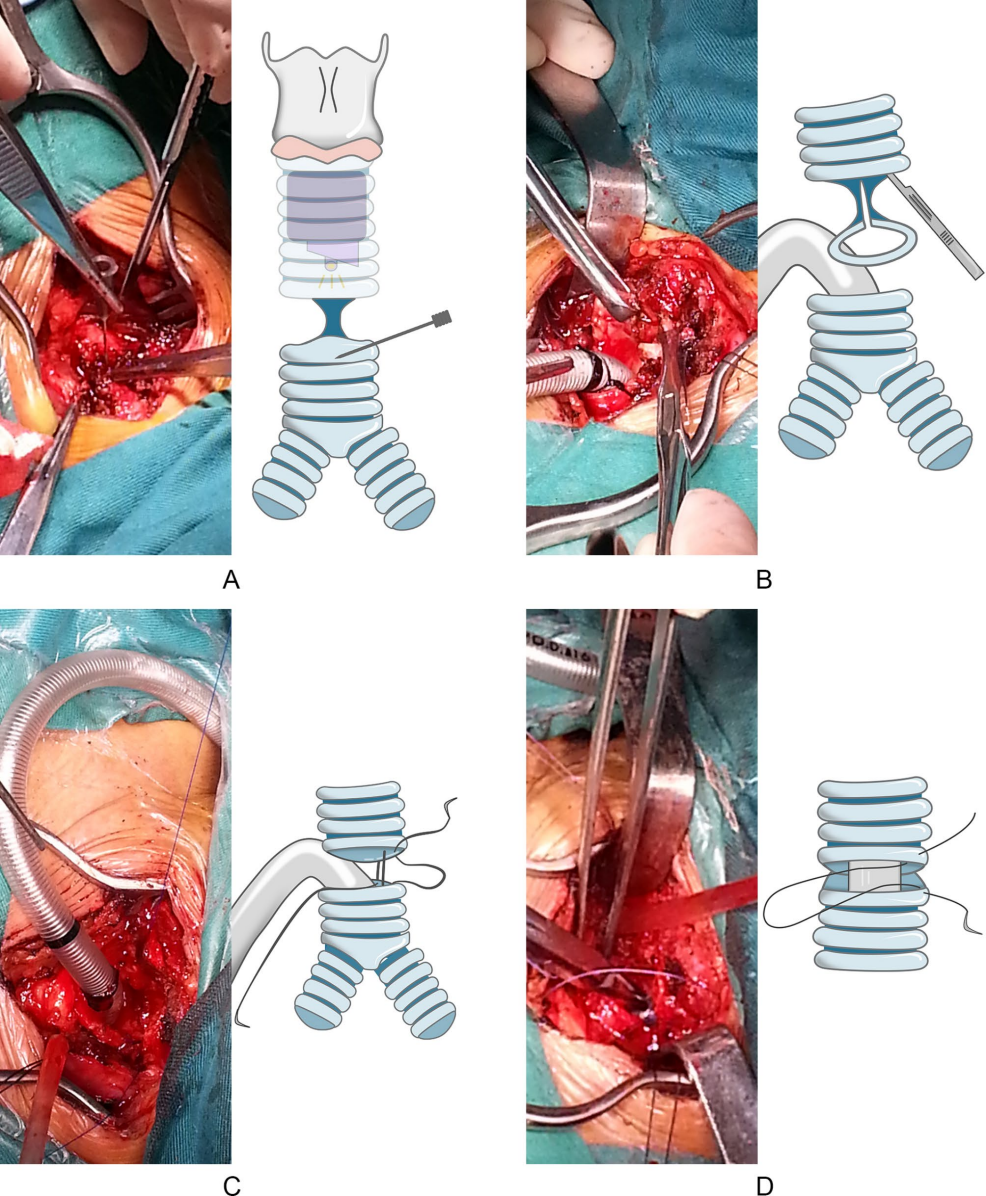
(**A**) Once the precise range of TS was identified, the anterior wall was incised at 0.5 cm distal from the lower edge of TS; (**B**) A backup tracheal catheter was inserted to distal trachea for MV, and isolation and separation of the stenosis was performed carefully to avoid the injury to recurrent laryngeal nerve; (**C**) The stenotic trachea was removed and the anastomosis of posterior wall of the trachea was performed continuously by with Prolene line; and (**D**) The distal backup tracheal catheter was removed and the proximal tracheal catheter was placed via the anastomosis into the distal trachea. The shoulder roll was removed and the anterior wall of trachea was sutured intermittently by vicryl sutures

The main steps are shown in Fig. 2. A syringe needle was positioned at the lower edge of stenosis which could be guided by fiberoptic bronchoscope so it could pass the stenotic lesion. The upper edge of stenosis as an alternative was identified by bronchoscope. Once the upper or lower edge was located, the anterior wall of trachea was incised longitudinally to identify the precise range of stenosis. The anterior wall was then incised 0.5 cm distal from the lower edge of the stenosis and a field tracheal catheter inserted to the distal trachea for ventilation. The trachea was transected 0.5 cm away from the upper edge of the stenosis and the stenotic trachea was removed. The anastomosis of posterior wall of the trachea was sutured continuously with a monofilament non-absorbable thread, e.g., Prolene 3 − 0 (Ethicon, Johnson-Johnson surgical technologies, USA), for convenience and a smooth edge. The distal field intubation was removed and the proximal tracheal catheter was placed via the anastomosis into the distal trachea. The shoulder roll was removed to flex the neck and the anterior wall of trachea was sutured interruptedly with absorbable sutures, e.g., Vicryl 3 − 0 (Ethicon, Johnson-Johnson surgical technologies, USA), which were respectively knotted with two terminals of Prolene and Vicryl to close the anastomosis. Air leakage was checked at the anastomosis site and then it was covered with sternocleidomastoid muscle flap, especially in the case with the anastomosis in vicinity of the brachiocephalic artery. Penrose drainage was performed peripheral to the anastomosis. Skin tissues of chin and superior chest wall were sutured to maintain neck anteflexion for seven days to reduce anastomosis tension and laryngoscopy was used to detect a potential recurrent palsy before extubation.

The relevant data of the 14 patients were retrospectively reviewed, and the operation time, blood loss, postoperative hospital stay, postoperative complications and follow-up were retrieved.

## Results

There was no intraoperative death. Preoperative treatment included tracheal intubation and MV in five cases, tracheotomy and MV in one case and oxygen inhalation in eight cases. Four patients underwent femoral artery-venous bypass surgery because severe stenosis with a diameter was less than or equal to 0.5 cm and anesthesia induction intubation was intolerable and unfeasible due to severe dyspnea and orthopnea. Neck incision, median sternotomy and right thoracotomy was conducted in nine cases, four cases and one case, respectively. End-to-end anastomosis of trachea was performed in 13 cases and the anastomosis between trachea and cricothyroid membrane was performed in one case. The length of resected trachea ranged from 1.5 − 4.5 cm with a mean of 1.67 ± 0.63 cm. Operation time ranged from 50 − 450 min with a mean of 142.8 ± 96.6 min and intraoperative hemorrhage ranged from 10 − 300 mL with a mean of 87.8 ± 83.6 ml. Follow-up period ranged from 5 to 43 months with a mean of 17.9 ± 10.6 months. Five cases were lost to follow-up. None of the patients had recurrent laryngeal nerve paralysis during postoperative follow-up. The clinical outcomes of the fourteen cases were shown in Table 2. Computed tomography (CT) confirmed the successful trachea reconstruction shown in Fig. 1B in 10 cases who were discharged uneventfully. Three cases were transferred into local hospitals for interventional therapies due to AS, shown in Table 2 and recovered successfully. However, Bronchopleurocutaneous fistula occurred in one patient after 6 days following trachea reconstruction who declined further treatment and was lost to follow-up.

**Table 2:** Clinical outcomes of 14 cases

Case	The length of resected trachea(cm)	Operation time(min)	Intraoperative hemorrhage(ml)	Postoperative MV time (hours)	Complications	Treatment of complications	Postoperative hospital stays(days)	Follow-up period(months)	Postoperative follow-up results and treatment
#1	2	130	170	20	Wound infection	Drainage	33	43	N.A.
#2	4	135	80	8	Wound infection	Drainage	26	21	N.A.
#3	4.5	120	100	18	Anastomotic hemorrhage	Hemostasis	16	19	Balloon dilations by bronchofiberscope due to AS
#4	4	160	50	63	Lung infection induced ARDS	Re-tracheotomy and MV	38	18	Extubation of Tracheostomy tube and tracheal stent was implanted due to AS
#5	2.5	110	20	14	Cholecystitis	Analgesia and anti-infection	17	16	N.A.
#6	4	450	300	168	Anastomotic fistula	Drainage and anti-infection	7	N.A.	The patient gave up treatment and lost to follow up.
#7	2.5	60	50	10	No	N.A.	23	12	N.A.
#8	1.5	70	30	18	No	N.A.	9	20	N.A.
#9	2	180	200	11	No	N.A.	22	20	N.A.
#10	2.5	125	80	16	No	N.A.	11	5	Treatment of argon plasma coagulation and cryotherapy due to AS
#11	3.5	50	20	5	No	N.A.	25	N.A.	N.A.
#12	3	115	10	0.5	No	N.A.	17	N.A.	N.A.
#13	3	120	100	4	No	N.A.	15	N.A.	N.A.
#14	2.5	174	20	6	No	N.A.	5	5	N.A.

**Abbreviations**: AS: anastomosis stenosis; MV: mechanical ventilation; and N.A.: Not available

Histological features of resected lesions included destruction of tracheal cartilage rings, proliferation of fibrous tissue and infiltration of chronic inflammatory cells.

## Discussion

The syndrome of TS can be caused by a variety of factors, but ischemic necrosis of the tracheal cartilage is believed to be the most common reason [9]. If the pressure of a balloon catheter exceeds capillary perfusion pressure of about 25 mmHg, the submucosal blood circulation can be occluded leading to ischemic necrosis of tracheal cartilage, fibroblast proliferation, granulation tissue formation, scar tissue contraction and final trachea stenosis [9, 10].Other risk factors include long term use of glucocorticoid or estrogen, respiratory failure, old age, obesity, hypertension and diabetes [11, 12].

Although tracheal resection and reconstruction is considered as the gold standard for TS therapy [3, 11, 13], interventional treatments will be the preferred choices under circumstances that include cases who are undergoing long-term MV where the balloon catheter and tracheal catheter can’t be withdrawn, the tracheal mucosa is unfit for anastomosis due to obvious inflammation or infection, the stenosis is longer than 50% of the trachea up to six cm and the length of webbed or membranous stenosis is less than one cm but with a complete tracheal cartilage ring.

Interventional therapies include balloon dilation, argon or laser cauterization and stent placement, which can’t always provide satisfactory long-term effects due to the high recurrence rates [6, 14, 15]. Hashemzadeh et al. [13]reported 39 cases of tracheal stenosis underwent repeated balloon dilatation, but all the cases finally received surgical treatments due to recurrence of scarring. If the efficacy of repeated dilatations was unsatisfactory, a tracheal stent can be used as an alternative, but it needs to be frequently replaced due to stimulation of granulation tissue. The incidence of stent migration and obstruction was reported to be 19.3% and 27.2% in the cases who received stent placement [16]. The short and webbed TS can be treated with laser cauterization, but tracheal cartilage rings can be accidentally injured [17]. Both Brigger et al. [10]and Grillo et al. [18] advocated that surgical treatment should be the initial choice for treatment of benign TS because preoperative interventional therapy may increase the incidence of postoperative complications and cause pathological changes resulting in difficulties for anastomosis. In this study, two cases underwent repetitive balloon dilation or laser cauterization but with unsatisfactory outcomes.

Anesthesia and MV are critical for subsequent manipulations. If the inner diameter of TS is less than 0.5 cm, rigid bronchoscopy was used for dilation prior to intubation of tracheal catheter [18]. If the diameter exceeded six mm, the catheter was inserted at the upper end of the stenosis to avoid injury to the tracheal mucosa caused by intubation [19]. In 10 cases, the tracheal catheter could be inserted at the proximal end of stenosis for MV, otherwise the lower edge was identified by a longitudinal incision of the anterior trachea wall and a field tracheal catheter was inserted across the lower end of stenosis for ventilation until the posterior wall anastomosis was completed. The field intubation was removed and the proximal tracheal catheter was inserted across the anastomosis site.

Considering the potential bleeding, ventilation dysfunction or asphyxia resulting from balloon dilation or rigid fiberoptic bronchoscope [20], especially in the patients suffered from the severe dyspnea and orthopnea, we finally chose to use temporary cardiopulmonary bypass in four cases, who were discharged from hospital after extubation, without intraoperative anoxia event. Similarly, cardiopulmonary bypass or ECMO support have been reported to yield good results [21], thus creating better surgery opportunities and conditions for patients with such severe dyspnea.

Tracheal sleeve resection and end to end anastomosis are feasible for resection length < 4 cm, however, age, height and other factors of patients deserve consideration. For short and elderly patients, resection of three cm is difficult to anastomosis. Complete division and isolation of the anterior and posterior walls ranging from thyroid cartilage to carina can reduce the tension of subsequent anastomosis. If the removed length exceeds 4.5 cm, infrahyoid laryngeal release or suprahyoid laryngeal release should be performed, requiring the resection of muscles and ligaments between hyoid bone and thyroid cartilage. Suprahyoid laryngeal release requires the resection of suprahyoid muscles and bilateral hyoid bone and these procedures can extend the trachea by 2.5 − 3 cm [22], but may affect laryngeal function leading to dysphagia and aspiration [18]. Laryngeal release is usually unnecessary for the majority of TS caused by tracheal catheter because the diameter of the balloon can’t exceed 2.5 cm [23]. In this study, the average length of tracheal stenosis and resection was 1.6 and 2.9 cm, respectively. In three cases, the length of resection was more than 4 cm (4.5 cm as the longest). However, laryngeal release was avoided because no excessive tension was detected during the anastomosis.

End-to-end anastomosis of trachea was the most critical and difficult procedure. There are three available anastomosis methods which include interrupted suturing of the posterior wall of trachea with absorbable suture and anastomosis of the anterior wall after replacement of tracheal intubation, continuous suturing of the posterior wall with nonabsorbable suture and anterior wall suturing after replacing the tracheal tube and continuous suturing of the posterior wall of the trachea with nonabsorbable suture, with intermittent suturing of the anterior wall with an absorbable suture. This study used third technique with hybrid sutures of both unabsorbable and absorbable suture. The former was used for anastomosis of the posterior wall because the continuous sutures were more convenient, smooth and easy as compared with interrupted sutures. Interrupted sutures were then used for anastomosis of the anterior wall to avoid the risk of fracture of the single unabsorbable stitch.

Tracheal resection and reconstruction remain challenging due to the technical complexity of tracheal anastomosis and postoperative complications. Anastomotic dehiscence, stenosis and fistula are relatively common anastomotic complications. Intraoperative anastomotic wrapping is a method to potentially reduce anastomotic complications, providing an additional layer of barrier to protect the anastomosis and separate it from arteries and other structures, leading to avoidance of fistula formation and promotion of anastomotic healing. In this study, anastomotic fistula occurred in one case due to poor exposure and tension of the anastomosis field, despite covering the anastomosis with a sternocleidomastoid muscle flap. For the patients with extended TS or deep TS, such as beneath innominate artery, a tracheal replacement/transplantation could be an alternative and after literature review and analysis, the thymopericardial fat flap could be a better alternative [24].

As a common postoperative complication, AS may be caused by severe mucosal pathological changes, such as proliferation of granulation and poor anastomotic techniques [13]. The proliferation of granulation was found postoperatively in 33% cases before 2000 [25], but improved with better sutures [18]. In 2013, Bagheri et al. [26] reported five cases suffered from AS in 20 cases (25%) following trachea reconstruction. In 2015, Ahn [5] reported three cases had AS among 18 cases (17.6%) with tracheal reconstruction. In this study, three cases had AS (21.4%) but subsequent treatments were successful. Overall, of the 14 cases in this study, ten cases (71%) had satisfactory curative effect and outcomes.

In summary, for surgical treatment of severe benign tracheal stenosis, the strategies of anesthesia, mechanical ventilation, identification of stenosis lesion, the “hybrid” sutures and postoperative anteflexion are very important to be optimized for successful postoperative recovery. However, we acknowledged the limitations of this study, including the small case number and absence of control group. New technologies and skills are supposed to be applied in the future to accumulate more experience.

## Data Availability

All data generated or analyzed during this study are included in this published article.

## Abbreviations

TS: Tracheal stenosis
MV: Mechanical ventilation
AS: Anastomosis stenosis
